# Time scale of glycation in collagen of bovine pericardium-derived bio-tissues

**DOI:** 10.1107/S2052252521010344

**Published:** 2021-10-28

**Authors:** Liberato De Caro, Alberta Terzi, Luca Fusaro, Davide Altamura, Francesca Boccafoschi, Oliver Bunk, Cinzia Giannini

**Affiliations:** a Institute of Crystallography, National Research Council, via Amendola 122/O, Bari 70126, Italy; b Department of Health Sciences, University of Piemonte Orientale, Novara Italy; c Tissuegraft srl., Novara Italy; d Paul Scherrer Institut, 5232 Villigen, PSI Switzerland

**Keywords:** computational modeling, structure prediction, materials modeling, imaging, structure determination, glycation, collagen

## Abstract

Glycosyl­ation is the process of combining one or more glucose molecules (or other monosaccharides) with molecules of a different nature (which are therefore glycosyl­ated). In biochemistry, glycosyl­ation is catalyzed by several specific enzymes, and assumes considerable importance since it occurs mainly at the expense of proteins and phospho­lipids which are thus transformed into glycoproteins and glycolipids. Conversely, in diabetes and aging, glycation of proteins is a phenomenon of non-enzymatic nature and thus not easily controlled. Glycation of collagen distorts its structure, renders the extracellular matrix stiff and brittle and at the same time lowers the degradation susceptibility thereby preventing renewal. Based on models detailed in this paper and with parameters determined from experimental data, we describe the glycation of type 1 collagen in bovine pericardium derived bio-tissues, upon incubation in glucose and ribose. With arginine and lysine/hy­droxy­lysine amino acids as the primary sites of glycation and assuming that the topological polar surface area of the sugar molecules determines the glycation rates, we modelled the glycation as a function of time and determined the glycation rate and thus the progression of glycation as well as the resulting volume increase.

## Abstract 2

Many pathological side-effects of diabetes and aging are directly linked to non-enzymatic glycation of collagen as an abundant, multi-purpose tissue in human beings. We describe a model that quantifies glycation and its structural effects as a function of sugar level (glucose and ribose) and time, based on SAXS and WAXS data.

## 1. Introduction   

Diabetes mellitus (DM) is a pathological condition characterized by a high blood glucose (hyperglycemia) and insulin concentration for a long period of time. The disorder is caused by a metabolic alteration named insulin resistance (IR), due to defects in insulin secretion (type 1 DM) or action (type 2 DM). This condition leads to several metabolic dysfunctions such as lipid accumulation, low-grade inflammation with foam cell development, tissue stiffening and atherosclerosis. The normal glucose concentration in blood is around 65–110 mg dl^−1^. When it increases to 126 mg dl^−1^ the condition is defined as impaired fasting glucose (IFG) which develops into DM for blood glucose concentrations over 126 mg dl^−1^ (American Diabetes Association, 2016 ▸). Among the complication of diabetes are alterations of micro and macro circulation, inducing multiple organ dysfunctions, and activation of pro-inflammatory pathways. The link between these pathological conditions and hyperglycemia are the advanced glycation end-products (AGEs) formed by the non-enzymatic crosslink reaction between small sugars, in particular glucose, fructose and ribose, and lipids and proteins. As glycation is a non-enzymatic reaction, it cannot be controlled, and the formation of AGEs is proportional to the blood sugar concentration. The multistep process of the formation of AGEs starts from the Maillard reaction: the addition of the electrophilic carbonyl group of reducing sugars with the free amino groups of the amino acid side chains such as lysine, hy­droxy­lysine or arginine. During this first step, a non-stable Schiff base is formed (adduct) and is subsequently stabilized in keto­amine, also known as Amadori’s product. When these molecules are dehydrated, oxidized or react with the side chains of amino acid residues forming crosslinks, the reaction becomes irreversible (Ahmed, 2005 ▸; Gkogkolou & Böhm, 2012 ▸). One of the principal targets of AGE accumulation is type I collagen, which is renewed every 10 years in skin, every 1–2 years in bones and it is slowly renewed in connectives.

Type I collagen is ubiquitous in our body as it is the main component of the fibrous network of tissues called extracellular matrix (ECM), which provides mechanical strength, stiffness and support to tissue functions and cell growth (Chen *et al.*, 2015 ▸). Collagen has a triple-helix structure, composed of two α1 (I) chains and one α2 (I) chain (Ramachandran & Kartha, 1955 ▸; Giraud-Guille, 1992 ▸; Bella *et al.*, 1995 ▸; Persikov *et al.*, 2004 ▸). The triple helix is characterized by two non-helical sections at both C and N-termini, and by the central helical section in which the typical triplet Gly *XY* is located. Note, while the Gly residue is highly conserved at the third position of the triplet, hidden at the center of the triple helix and preserving the packing of the molecule, the amino acids in the *X* and *Y* positions (often proline and hy­droxy­proline) are exposed on the molecular surface for the steric interaction with other molecules. Triple-helical stabilization is also permitted by the formation of hydrogen bonds between the NH group of Gly in the backbone of a chain and the CO group of the *X* amino acid on the neighboring chain, or water mediated. Indeed, the stabilization role of the hydration cylinder that surrounds each molecule is well known. It allows for the assembly of molecules in a supramolecular staggered structure, with a meridional periodicity *d*_M_ ≃ 65 nm along the fiber axis and a lateral equatorial distance *d*_E_ ≃ 1.5 nm, depending on the hydration state of the structure, perpendicular to the fiber axis (Orgel *et al.*, 2000 ▸, 2001 ▸). The extent of glycosyl­ation – the physiological enzymatic addition of sugars to the protein, – affects the distance between the centers of two neighboring molecules (enzymatic crosslinking). Collagen stabilization is also guaranteed by the formation of enzymatic crosslinks between nanofibrils. Mass spectrometry studies revealed that both glycation (non-enzymatic) and enzymatic crosslinks occur at the amino acidic lysine residue. Precisely, the reactions compete for the same reaction site, *i.e.* the ɛ amine of the lysine side-chain. Indeed, Hudson *et al.* (2018 ▸) observed a decrease of enzymatic crosslinks not only on the surface, but also in the inner part of the collagen fibrils and, at the same time, an increase of the non-enzymatic ones. Furthermore, it was observed that both reactions also occur on hy­droxy­lysine and arginine residues of the triple helices. In particular, it was found that, during collagen growth in tendons, aldehydic lysine and hy­droxy­lysine target specific lysine and hy­droxy­lysine residues in the helical domain of neighboring molecules (Lys87 and Lys930, Hys 933) for the formation of intermolecular crosslinks. The same residues are preferred targets of glucose and ribose 5P in glycation; indeed, the non-enzymatic glycation mainly occurs on lysine, hy­droxy­lisine and arginine residues of the triple helices. The glycation of lysine alters not only the length of its side chain, because of the sugar addition, but also the charge distribution of the lateral chains. This leads to the disruption of molecular organization, an altered fibrillary packing and interaction with the other components of the ECM, and modification of fibrillary axial banding. The *d*_M_-period is stabilized by charge–charge interactions, between lateral positive chains of adjacent amino acids, but the formation of neutral or negative adducts, or crosslinks, can modify the charges thereby destabilizing the structure (Hadley *et al.*, 1998 ▸, 2001 ▸). Thus, the fibrillary disorder occurs along the fibril axis and at the stagger position. The result of these alterations is a less elastic collagen, with an increased stiffness and easy rupture, *i.e.* a brittle ECM. The glucosepane (Sell *et al.*, 2005 ▸) is the most abundant and relevant AGE in collagen-rich tissues, as it was found to increase by 1000 times in diabetics with respect to non-pathological tissues. It is a characteristic crosslink between lysine, glucose and arginine. Molecular simulation studies demonstrated that the intramolecular presence of glucosepane increases the hydration but decreases the helical packing. It rearranges its molecular structure to maximize the polar contact: it settles down its polar fraction at a distance of about 0.25 nm from hy­droxy­proline and 0.27 nm from proline. This causes the loss of packing of triple helical α-chains in the final triple helix, and the alteration of the intra-molecular packing. The nanometric collagenic organization appears more cross-linked but with less molecular confinement in the fibrillary structure, and despite the wider helical packing, there is a decrease of degradation susceptibility of collagen because of the high number of crosslinks. Gualtieri *et al.* (2014 ▸, 2017 ▸) highlighted that glucosepane can be found at both the intra and the intermolecular positions and that the reaction distance between two amino acid residues (lysine and arginine) for its formation must be in the range 2.6–3.8 Å. Note that the lysine and arginine of 14 couples are close enough for the formation of 6 intramolecular and 8 intermolecular glucosepane crosslinks. Furthermore, most of the lysine involved in the formation of glucosepane are also binding sites for eparine, proteoglycans and integrin, whereas Arg789, also involved in the formation of AGEs, is closer to the MMP-1 binding site. Thus, the non-enzymatic crosslink at this arginine aids in decreasing the degradation susceptibility of collagen, sterically inhibiting the MMP-1 binding and activity.

Despite the obvious relevance of the glycation of collagen, we are not aware of widely accepted quantitative and verified models for the progression of glycation as a function of sugar concentration and time. Furthermore, quantitative information on the differences between the biologically relevant sugars appears to be scarce as well. Here, we describe a model for the non-enzymatic glycation of collagen as a function of time. Based on our own experimental data available for up to a duration of 90 days, we determine the free parameters in this model to fit the experimental observations. Based on this glycation model, glycation rates for much longer durations of up to 10 years are estimated. These could be verified experimentally, for example, using the tissue model system on which the experimental part of this study is based.

## 2. Results   

**Figure 1:**
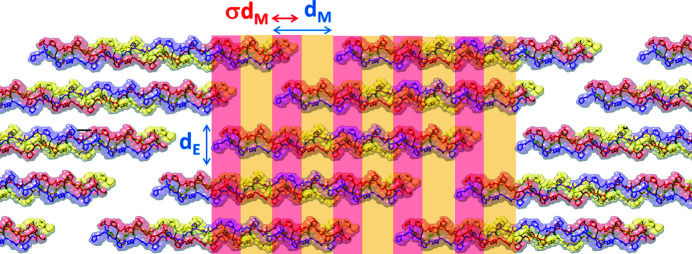
Schematic of the staggered repetition unit of the triple-helix collagen structure. The fiber axis is along the arrow indicating dense σ*d*_M_-wide regions with five nanofibrils overlapping alternately with less dense (1 − σ)*d*_M_-wide regions of one gap and four nanofibrils overlapping.

The length of a single triple-helix molecule (nanofibril about 306 nm long and 1.5 nm wide) is not an integer multiple of the meridional periodicity *d*_M_ ≃ 65 nm. Nanofibrils are assembled in a supramolecular staggered repetition unit, with five nanofibrils packed at *d*_E_ ≃ 1.5 nm equatorial lateral distance, as schematically shown in Fig. 1 ▸. This unit is formed by a σ*d*_M_-wide region (σ < 1), characterized by a higher electron density value, resulting from the overlap of the five nanofibrils fractions (overlap region, marked by the red bands), and a complementary region (gap region, marked by the yellow bands), (1−σ)*d*_M_ wide, which contains one gap and the four nanofibrils fractions.

Decellularized bovine pericardia have been used as collagen tissue and kept in a water solution mixed with sugars at increasing concentration from 0 to 40 mg ml^−1^ for different incubation times, from 0 to 90 days (Giannini *et al.*, 2019 ▸, 2021 ▸), and for different type of sugars: ribose, glucose and galactose. Scanning small- and wide-angle X-ray scattering data (SAXS, WAXS) were collected at the cSAXS beamline of the Swiss Light Source (Bunk *et al.*, 2009 ▸). Experimental details can be found in the literature (Giannini *et al.*, 2019 ▸; Bunk *et al.*, 2009 ▸).

**Figure 2:**
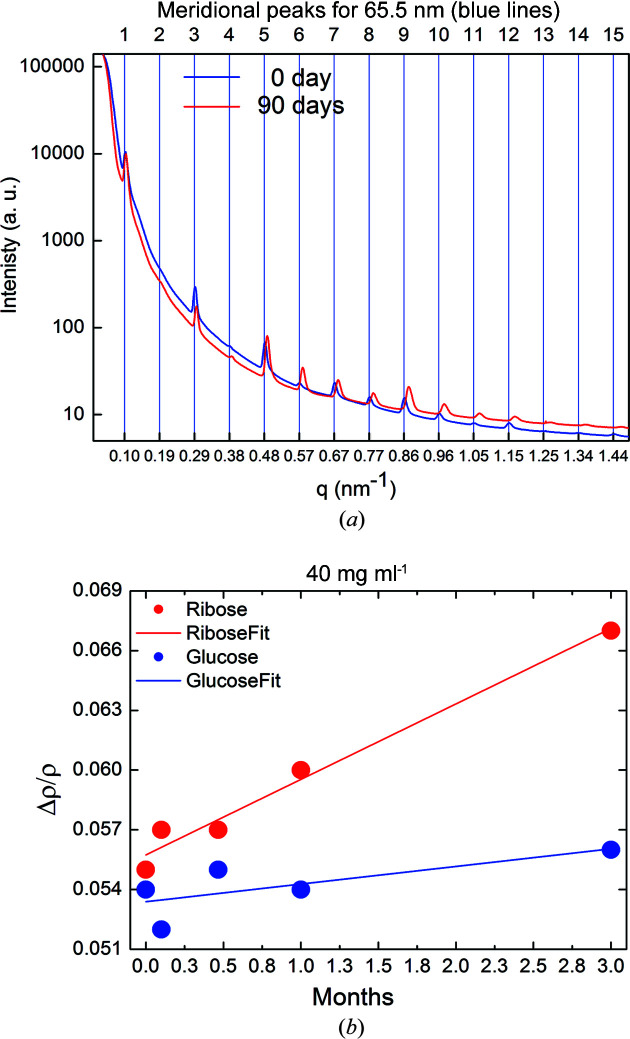
(*a*) SAXS profiles for collagen incubated in a ribose solution of 40 mg ml^−1^ at 3 (blue) and 90 days (red) (Giannini *et al.*, 2021 ▸). (*b*) Relative electron density difference (Δρ) determined for the collagen samples in solution with 40 mg ml^−1^ ribose (red dots) and glucose (blue dots), as a function of the incubation time, together with a linear fit of the data (straight lines).

SAXS and WAXS techniques allow us to probe collagen at different length scales from tens to fractions of nanometres and to achieve structural, microstructural and morphological characterization in a non-invasive way and without charge build-up. Precisely, for the length scales of interest here, WAXS keeps track of the intermolecular *d*_E_ ≃ 1.5 nm equatorial lateral distance (Giannini *et al.*, 2021 ▸), and SAXS allows us to probe the supramolecular electron density distribution due to the meridional periodicity *d*_M_ ≃ 65 nm along the fiber axis, which produces a diffraction profile with a series of equispaced Bragg peaks (see Fig. 2 ▸). X-rays are scattered by the electron density ρ of matter, and if the zero-order diffraction is not measured as a result of inherent technical difficulties, then the average electron density value ρ_ave_ is unknown. For this reason, SAXS permits probing of only relative electron density differences (Δρ) between overlap and gap regions, given as a fraction of ρ_ave_.

Fig. 2 ▸ (*a*) shows the azimuthally integrated SAXS profiles collected on bovine pericardium tissues kept in a 40 mg ml^−1^ ribose solution for 3 (blue) and 90 days (red) (Giannini *et al.*, 2021 ▸). The relative electron density difference Δρ, derived from SAXS data, was found to increase linearly, as a function of the incubation time, from 3 to 90 days (3 months), as displayed in Fig. 2 ▸(*b*) for ribose (red dots) and glucose (blue dots) at 40 mg ml^−1^, and reported in Table 1 ▸ for ribose and Table 2 ▸ for glucose. The solution was refilled in the investigated sample every week to avoid dehydration. Therefore, the concentration of the solution can be assumed constant as a function of time, and identical inside and outside the molecule. For glucose, the relative electron density variations after 90 days (Δρ_f_) with respect to the initial values at 0 days (Δρ_i_) are quite small, given by Δρ_f_ − Δρ_i_ = 0.002 ± 0.002. Conversely, for ribose we observe a significantly larger change: Δρ_f_ − Δρ_i_ = 0.012 ± 0.002.

The experimental results in Table 1 ▸ show that, after 90 days in the presence of a solution of 40 mg ml^−1^ ribose in water, the equatorial distance *d*_E_ between collagen nano-fibrils increases from 1.51 ± 0.02 to 1.69 ± 0.02 nm, whereas the meridional periodicity *d*_M_ of the collagen electron density decreases from 65.5 ± 0.1 to 64.4 ± 0.1 nm. Conversely, for a glucose solution (40 mg ml^−1^) we found constant values for the *d*_M_ meridional period and a smaller variation of the equatorial value *d*_E_ (Table 2 ▸) compared with the variations measured for the ribose solution (Table 1 ▸) at the same concentrations. Additionally, for the structures under investigation, we found σ = 0.475 ± 0.005 for all samples, *i.e.* constant length of gap and overlap region along the collagen fibers for both ribose and glucose solutions (Giannini *et al.*, 2021 ▸). The variations of the relative electron density reported in Table 1 ▸ are expected to be related to glycation processes (Bailey *et al.*, 1995 ▸; Hudson *et al.*, 2018 ▸; Hadley *et al.*, 1998 ▸, 2001 ▸; Madhurapantula & Orgel, 2017 ▸) as we will discuss.

**Figure 3:**
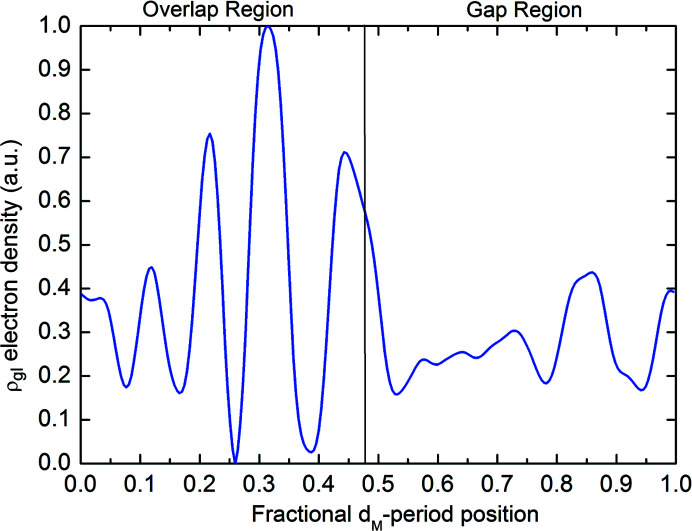
Electron density profile derived by the Fourier difference phasing carried out on the SAXS patterns in Fig. 2(*a*) ▸.

The Fourier difference synthesis, computed from the SAXS patterns (explained in the Experimental[Sec sec5]) produces the relative electron density shown in Fig. 3 ▸. The resolution of the SAXS data is limited by the maximum measured scattering angle, corresponding to the 15th reflection in Fig. 2 ▸(*a*). Therefore, the spatial resolution of the ρ_gl_ profile is 65 nm/15 = 4.3 nm.

**Figure 4:**
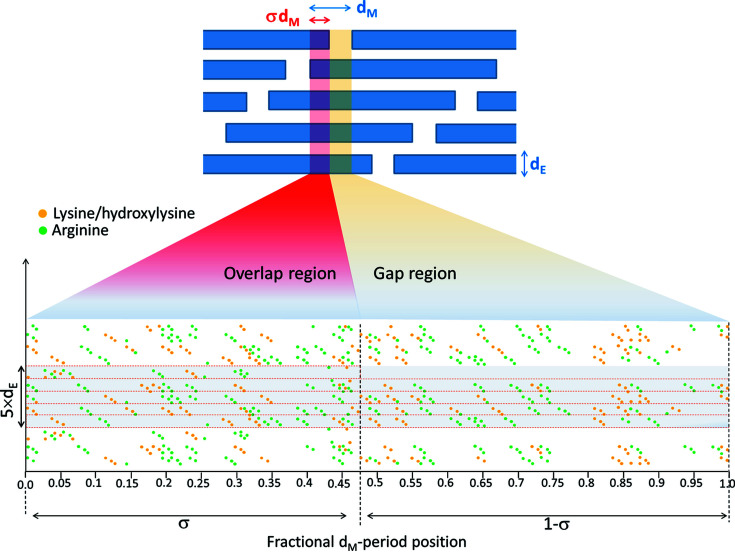
Top: schematic (not to scale) of the collagen staggered structure. Bottom: linearized projection of the 3D triple-helix collagen residues, with the lysine/hy­droxy­lysine (orange bullets) and arginine (green bullets) amino acid positions shown as a function of the fractional *d*_M_-period coordinate.

The electron density profile shown in Fig. 3 ▸ is named ρ_gl_, being ascribed to glycation processes occurring near the arginine and lysine amino acids of the collagen structure (Gautieri *et al.*, 2014 ▸; Madhurapantula & Orgel, 2017 ▸). A magnification of the staggered repetition unit is shown in Fig. 4 ▸ (bottom), which shows a 2D projection of the triple-helix collagen residue, the lysine/hy­droxy­lysine (orange bullets) and arginine (green bullets) amino acids positions. This arrangement is obtained by projecting the linearized 3D triple-helix collagen structure (Madhurapantula & Orgel, 2017 ▸), reporting the residue positions as fractional coordinates of the *d*_M_-period. The whole staggered repetition unit contains about 1050 residues, fractioned in about 4.5 parts (average value between the 5 staggered nanofibril fractions of the overlapping region and 4 of the gap region); 1050 divided by 4.5 gives 233 residues, and 65.5 nm divided by 233 gives 0.28 nm, which measures the rise of the collagen helix per residue.

Thus, the 2D map of Fig. 4 ▸ requires a step finer than 1/233 in the sampling of the fractional *d*_M_-period coordinate values, ranging from 0 to 1. To visualize the amino acids in this 2D map (with bullets *n*-pixels large) we need a sampling finer than 1/(*n* × 233). We used a sampling of 1/1930, for *n* = 4. In this way the fractional-coordinate resolution, along the *d*_M_-period, is suitable to visualize all residues in a planar quantitative arrangement. We used the same pixel size both along the *d*_M_ horizontal and the *d*_E_ vertical direction.

**Figure 5:**
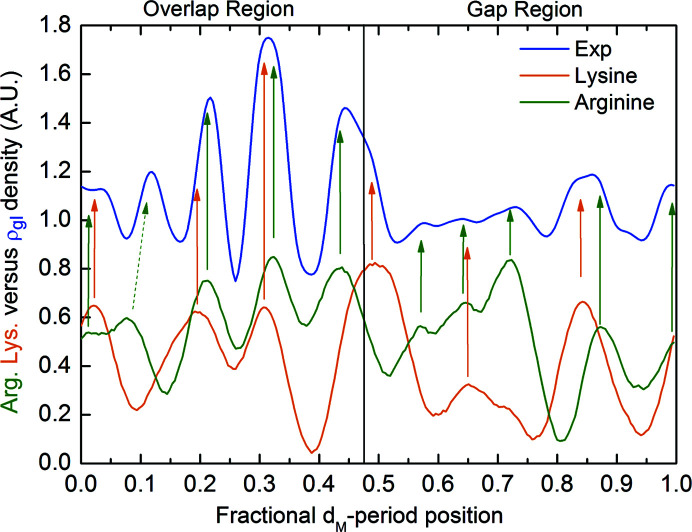
Linear densities of lysine/hy­droxy­lysine and arginine amino acids as a function of the fractional *d*_M_-period coordinate along the collagen fibril versus the electron density obtained by the Fourier difference phasing.

The electron density of all arginine (green bullets) and lysine/hy­droxy­lysine (orange bullets) amino acids of Fig. 4 ▸ (bottom part) has been summed along the lateral *d*_E_ axis and is shown in Fig. 5 ▸ as a function of the fractional *d*_M_-period coordinate. The green curve refers to the linear densities of the arginine and the orange curve of the lysine/hy­droxy­lysine amino acids.

These curves are compared with the experimentally determined electron density ρ_gl_ already shown in Fig. 3 ▸ as a blue curve. Colored vertical arrows highlight the interesting correspondence between the peaks of the lysine/hy­droxy­lysine and arginine amino acids’ linear densities and ρ_gl_, except for one peak, denoted with an oblique dashed arrow on the left of the figure. This clearly demonstrates that ρ_gl_ obtained by the Fourier difference phasing is related to the lysine/hy­droxy­lysine and arginine residues’ position within the collagen structure.

**Figure 6:**
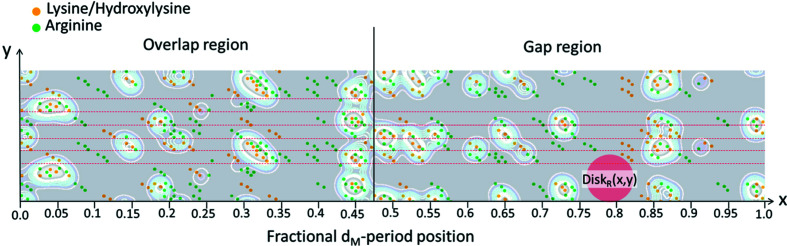
Likelihood for glycation. Shown is the 2D mapping of one period of the collagen structure (as in Fig. 4 ▸). The regions of the staggered repetition unit are denoted by dashed red lines. Superimposed is the probability function *D*_arg–lys_(*x*,*y*). The contours reveal where arginine (green bullets) and lysine/hy­droxy­lysine (orange bullets) are close to each other. At these positions the likelihood for glycation is high. The experimental resolution is indicated via a red disk, Disk_R_(*x*,*y*).

Ribose-mediated cross-links between arginine and lysine/hy­droxy­lysine residues are more probable where these amino acids are close to each other. To estimate a probability for glycation as a function of the fractional *d*_M_-period coordinate, we assume that the glycation is proportional to the product of the distributions of the amino acids, *D*_arg_(*x*,*y*) × *D*_lys_(*x*,*y*), see Fig. 6 ▸, where *x* and *y* are the horizontal (fiber axis) and vertical (lateral direction) coordinates, respectively. This product gives a likelihood of proximity of arginine and lysine/hy­droxy­lysine amino acids. The electron density due to glycation processes (blue curve of Fig. 5 ▸) is larger where more sugar molecules are cross-linked to these amino acids, *i.e.* where the likelihood of proximity of arginine and lysine/hy­droxy­lysine is larger. To take the experimental resolution into account, we blurred the projected distribution of the two amino acids shown in Fig. 4 ▸ by convoluting with a circular disk Disk_R_(*x*,*y*) of width *R* equivalent to the 4.3 nm spatial resolution of the experimental ρ_gl_ profile: *D*_arg–lys_(*x*,*y*) = *D*_arg_(*x*,*y*)⊗Disk_R_(*x*,*y*) × *D*_lys_(*x*,*y*)⊗Disk_R_(*x*,*y*). Thereby, we obtain a 2D function with maxima where many pairs of arginine and lysine/hy­droxy­lysine amino acids are at close distance. The maxima of *D*_arg–lys_(*x*,*y*) are shown in Fig. 6 ▸ as a contour plot, superimposed onto the 2D amino acid map shown in Fig. 4 ▸.

**Figure 7:**
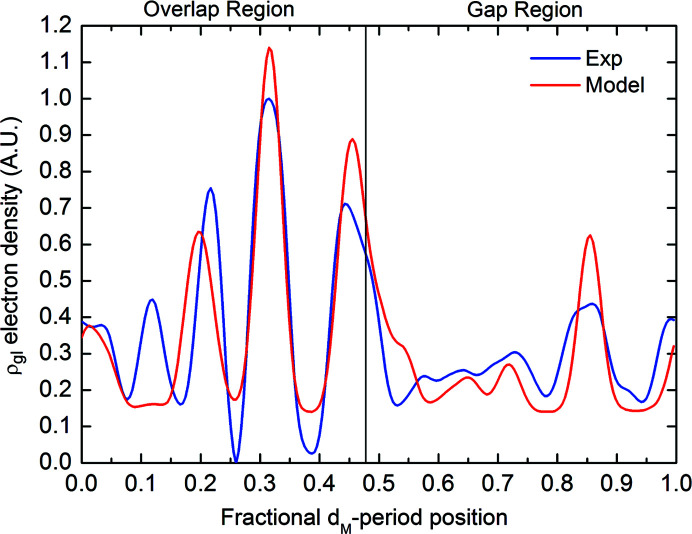
*D*_arg–lys_(*x*) function (red curve) versus ρ_gl_ obtained by the Fourier difference synthesis of the experimental SAXS data (blue curve) as a function of the fractional *d*_M_-period coordinate. Both functions were normalized to the same integral value.

Considering the 4.3 nm spatial resolution of our SAXS measurements, uncertainties of even a few Ångstroms in the 2D mapping of the distances between these amino acids should not influence the calculation of the function *D*_arg–lys_(*x*,*y*). Its integration in the *y* (vertical) direction of Fig. 6 ▸ leads to a 1D function, *D*_arg–lys_(*x*), labeled ‘Model’ in Fig. 7 ▸. It depends only on the fractional *d*_M_-period coordinate *x*.

Fig. 7 ▸ compares *D*_arg–lys_(*x*) to the experimentally derived ρ_gl_. The two curves have been renormalized to have the same integral. Their correlation, which is independent of any normalization and any multiplicative constant, is quite high, namely 0.86. Thus, we can conclude that our Fourier difference synthesis of SAXS data allows us to localize the ribose molecules within the collagen structure and that the glycation processes occur close to the maxima of the function *D*_arg–lys_(*x*,*y*) previously described.

Changes in the tertiary and quaternary structure of collagen, here neglected, also caused by glycation processes, probably hinder achieving higher correlation values.

## 3. Discussion   

### 3.1. Number of glycation crosslinks per *d*_M_-period along the collagen fibril   

**Figure 8:**
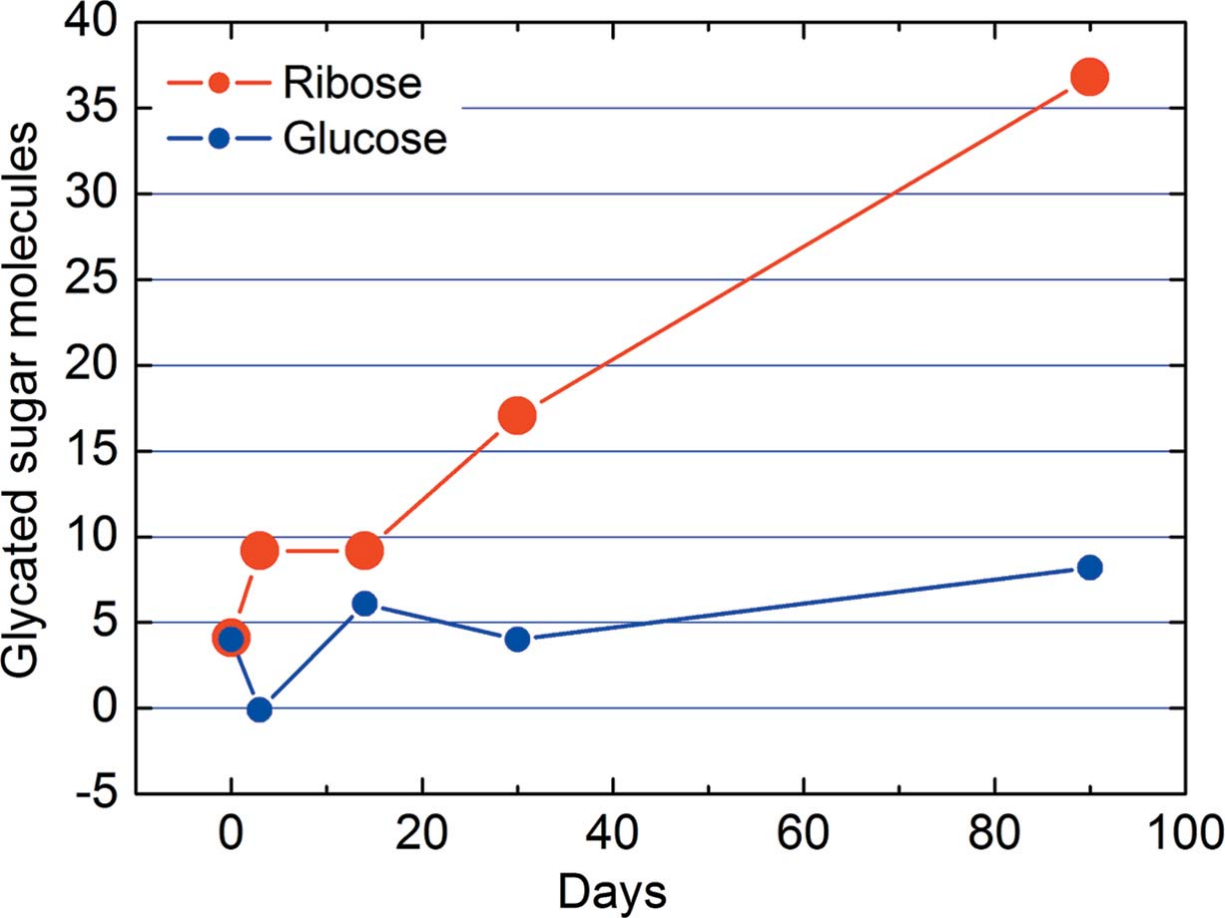
Number of sugar molecules involved in the glycation of one collagen repeat unit as predicted by Equation (S9) for a solution of either 40 mg ml^−1^ ribose (red) or glucose (blue), in water.

Collagen has a molar mass of about 300 000 g mol^−1^, which means *N*_e_ ≃ 160 000 electrons per molecule (Gelse *et al.*, 2003 ▸; León-López *et al.*, 2019 ▸). The electron density increment relative to the average electron density for collagen immersed in a ribose solution of 40 mg ml^−1^ for 90 days was (Δρ_f_ − Δρ_i_)/ρ_ave_ = 0.012 ± 0.002, as derived by SAXS measurements (Giannini *et al.*, 2021 ▸). This value multiplied by *N*_e_ gives about 1900 ± 300 electrons which, divided by 80 (the number of electrons in a ribose molecule) gives 24 ± 4 molecules. As demonstrated in Fig. 2 ▸(*b*), we are still far from saturation after 90 days of incubation. Indeed, after three months, the increment of the relative electron density is still linear as a function of the incubation time. Thus, we expect that saturation will occur for a larger number than this rough estimate of 24 glycated ribose molecules. A more detailed calculation of the number of sugar molecules *N* involved in glycation of the collagen staggered repetition unit is estimated by Equation (S9) of the supporting information and plotted in Fig. 8 ▸. For a concentration of 40 mg ml^−1^ after 90 days of incubation, the number of sugar molecules is *N* = 36.8 ± 5.7 for ribose and *N* = 8.2 ± 5.7 for glucose.

We could argue that the 36.8 ± 5.7 ribose molecules estimated based on our data could be just those involved in glycation processes between these lysine/hy­droxy­lysine and arginine pairs. This value should be compared with the 86 lysine–arginine pairs that are found closer than the cutoff distance of 0.5 nm in at least one frame of the molecular dynamics trajectory simulations, as reported by Gautieri *et al.* (2017 ▸). This comparison seems to indicate that a 90 day incubation of collagen fibers in a ribose solution of 40 mg ml^−1^ is sufficient to permit about half of all the possible glycation processes to happen in the structure, confirming that we are still far from saturation, as it can be deduced from Fig. 2 ▸ (*b*). For glucose, after 90 days we found 8.2 ± 5.7, a value about 4–5 times smaller than for ribose.

### 3.2. Equatorial and meridional period variations as a function of the incubation and thus glycation time   

**Figure 9:**
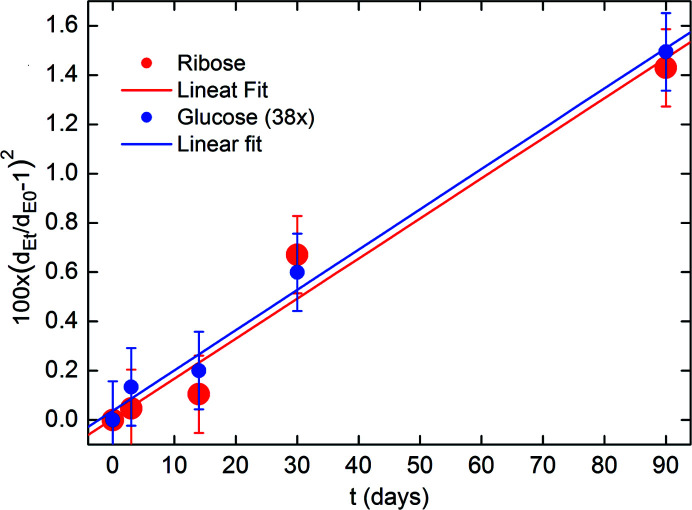
Squared relative variation of the equatorial period *d*_E_ at the incubation time *t*, denoted by *d*_Et_, with respect to the value at *t* = 0, denoted by *d*_E0_. This ratio is a measure for the variation of the equatorial in-plane areal density of collagen molecules as a function of the incubation time. Please note that the glucose data have been multiplied by a constant factor of 38.

The equatorial lateral areal density of the collagen molecules as a function of the incubation time is shown in Fig. 9 ▸. We plotted the relative variation (*d*_Et/_ *d*_E0_−1)^2^ of the equatorial period *d*_E_ at the incubation time *t*, denoted by *d*_Et_, with respect to the value at *t* = 0, denoted by *d*_E0_. We note a linear dependence of (*d*_Et_/*d*_E0_ − 1)^2^ for both ribose (red) and glucose (blue). The glucose data have been multiplied by a constant factor, equal to *f* = 38, to rescale the experimental points and nearly overlap with those obtained for the ribose solution. The two fitting straight lines have the same slope: Δ*d*_E_ = 1.63 × 10^−4^ ± 0.09 × 10^−4^ days^−1^ for ribose and 1.63 × 10^−4^ ± 0.2 × 10^−4^ days^−1^ for the 38 × (*d*_Et_/*d*_E0_−1)^2^ glucose data. Therefore, the actual slope of the fitted straight line of the (*d*_Et_/*d*_E0_−1)^2^ glucose data is 1/38 of 1.63 × 10^−4^ ± 0.2 × 10^−4^, *i.e.* equal to 0.043 × 10^−4^ ± 0.005 × 10^−4^ days^−1^.

Therefore, the in-plane packing areal density of collagen molecules varies linearly with incubation time for both ribose and glucose, but with a time scale factor *f* = 38 smaller for glucose with respect to ribose (see the supporting information for details).

**Figure 10:**
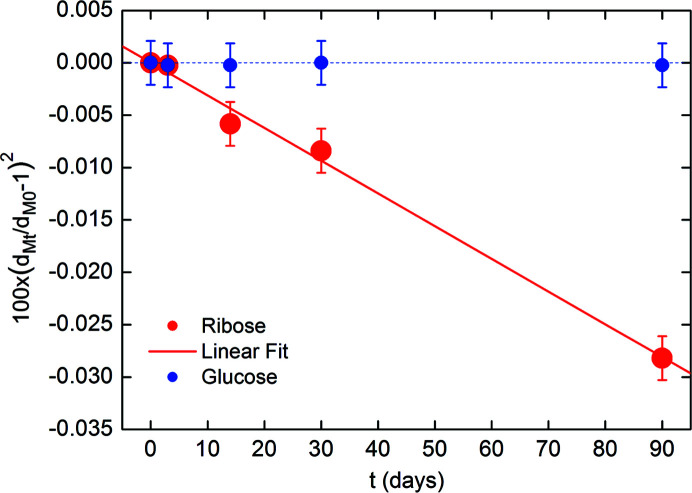
Squared relative variation of the meridional period *d*_M_ at the incubation time *t*, denoted by *d*_Mt_, with respect to the value at *t* = 0, denoted by *d*_M0_. The continuous red line is the linear fit of the ribose data (red), the horizontal dashed line is only reported as a guide for the glucose data (blue).

Moreover, the density of the collagen molecules versus the incubation time varies for modifications of the structure along the molecule axis, as shown in Fig. 10 ▸. Here we have plotted the (*d*_Mt_/*d*_M0_−1)^2^ relative variation of the *d*_M_ meridional period at incubation time *t*, denoted by *d*_Mt_, with respect to the value at *t* = 0, denoted by *d*_M0_. This plot aims to derive the density variation of collagen molecules, as a function of the incubation time, along the fiber axis. We note a linear dependence of (*d*_Mt_/*d*_M0_−1)^2^ for ribose (red). For glucose (blue) no variations have been detected experimentally, within the experimental errors, even after 90 days of incubation. The continuous red line is the linear fit of the ribose data (red), the blue dashed line is the horizontal axis.

The fitting line of the ribose data, shown in Fig. 10 ▸, has the slope Δ*d*_M_ = −3.1 × 10^−6^ ± 0.15 × 10^−6^ days^−1^. Thus Δ*d*_M_ is two order of magnitude smaller than Δ*d*_E_. Therefore, the change in volume of the collagen fibrils Δ*V* = (Δ*d*_E_ + 1) × (Δ*d*_M_ + 1) − 1 upon incubation in ribose is largely determined by the change in the equatorial area.

### 3.3. Time scales of glycation crosslinking   

The AGEs estimated accumulation percentage *R*_AGE_ is about 3.7% per year (Gkogkolou & Böhm, 2012 ▸). Therefore, the AGEs estimated accumulation is about 0.01% per day, under the roughly simplifying assumption of linear progression over the whole time range requiring a time as long as 10 000 days (27 years) to reach saturation. This value, however, is dependent on the particular sugar involved in the glycation processes. Indeed, as reported above, we observed different glycation rates for different sugar molecules. Testing the hypothesis that the topological polar surface area (PSA) of the sugar molecules determines the glycation rates, we define a simple model for glycation rates, to be compared with the experimental data,

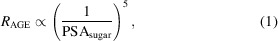

since the number of laterally staggered collagen molecules per in-plane unit cell is five (see Fig. S1 of the supporting information).

For this model, we introduce PSA_nat_ as a reference topological PSA describing the effective space available between collagen molecules, inside their ‘native’ in-plane packing, to accommodate sugar molecules. Thus, we should expect that the glycation rate will depend on the inverse of the ratio of the sugar PSA with respect to the PSA_nat_ value, raised to the power of five. As shown in the supporting information, the fraction of in-plane space between the collagen molecules is *w*_nat_ = 0.255 ± 0.04. Thus, for each of the five collagen molecules we have a fraction of available space equal to 0.051 ± 0.008. The size of the native in-plane unit cell can be estimated by the model discussed in Fig. S1, by evaluating the area of the parallelogram with red dashed lines, obtaining 21.9 ± 0.15 nm^2^. This value is about 26% larger than the native value (see supporting information). Therefore, a surface of 17.4 ± 0.12 nm^2^ could be associated with the unit cell of the native structure. This result allows us to estimate PSA_nat_ to be 0.051×17.4 nm^2^, *i.e.* PSA_nat_ = 89.0 ± 15.0 Å^2^.

Np = 86 pairs of arginine and lysine/hy­droxy­lysine amino acids (Gautieri *et al.*, 2017 ▸) are available for glycation processes. If all these pairs are glycated no further glycation processes can happen. Thus, based on the assumption (American Diabetes Association, 2016 ▸) and for one molecule of sugar per staggered repetition unit, the probability of glycation in the collagen fibril within the incubation *t* can be defined as

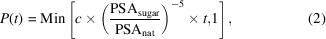

where *c* is a fitting constant of the model, whose value should be compared with the 0.037 per year value estimated by Gkogkolou & Böhm (2012 ▸).

In the presence of *N*_s_ rather than only one molecule of sugar in the available fraction of volume *w* [given by the product of Equations (S10) and (S18) for ribose, and by the product of Equations (S10) and (S19) for glucose], we need to consider that, in any of the sites of an arginine and lysine/hy­droxy­lysine amino acid pair any of these sugar molecules could give rise to a glycation process. If it is not the first sugar molecule giving rise to a glycation – probability of this event is 1 − *P*(*t*) – it could be the second molecule, leading to a contribution of [1 − *P*(*t*)]*P*(*t*) to the total probability and so on, for the third, fourth, ..., molecule. Therefore, the total probability for *N*_s_ sugar molecules can be computed as follows,

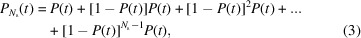

where

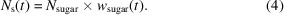

Here, *N*_sugar_ is defined in Equation (S10) as the number of sugar molecules contained in a cylindrical volume of solution comparable to the volume of a collagen staggered repetition unit, and *w*_sugar_ is defined in Equations (S18) and (S19) as the fraction of space between the (assumed to be cylindrical) collagen molecules within the 2D unit cell.

Finally, the whole number of glycations *N*_glyc_ can be computed by multiplying Equation (3) by the number of available arginine and lysine/hy­droxy­lysine pairs *N*_p_:

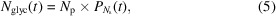

(Ramachandran & Kartha, 1955 ▸).

**Figure 11:**
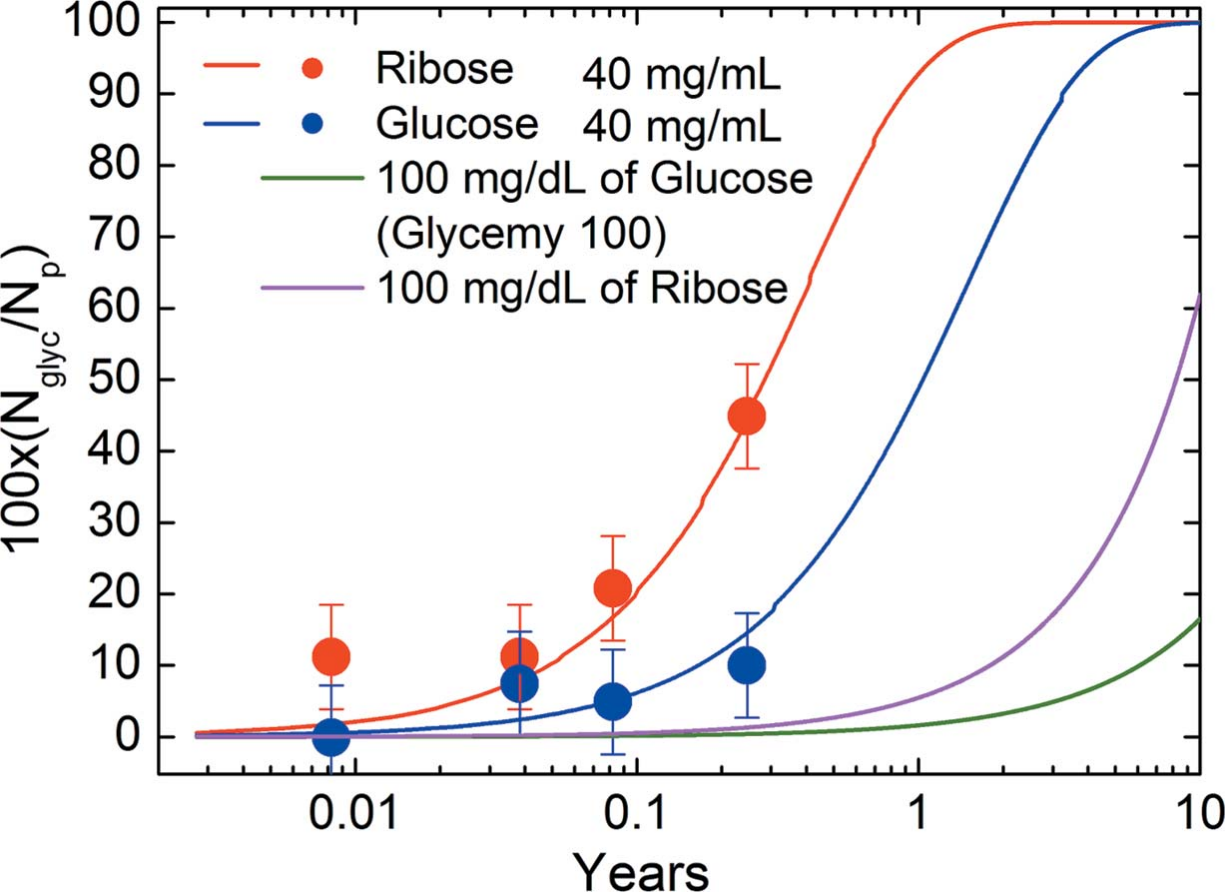
Percentage of glycations, predicted by Equation (5), as a function of the incubation time, calculated using the glycation probability according to Equation (3).

Fig. 11 ▸ shows the predictions of the model just described in comparison with the experimental results for ribose (red) and glucose (blue) at a concentration of 40 mg ml^−1^. The only free parameter of the theory is *c*, determined by imposing that the theoretical model must fit the number of ribose glycated molecules after 90 days of incubation as given by our data analysis shown in Fig. 8 ▸. The value obtained by the comparison with experimental data is *c* = 0.069 ± 0.001 per year. Thus, for ribose the AGEs estimated accumulation value per year is *R*_AGE,ribose_ = *c*[(PSA_ribose_)/(PSA_nat_)]^5^ = 0.064 ± 0.031 per year. For glucose we found that minimum and maximum values allowed by the experimental errors lead to *R*_AGE,glucose_, ranging between 0.01 and 0.053 per year. For both ribose and glucose the obtained values are comparable to that estimated by Gkogkolou & Böhm (2012 ▸), *i.e.* 0.037 per year, even if ribose glycation rates per year are about 2.4 faster than glucose ones. As the curves in Fig. 11 ▸ and 12 are not linear functions, this difference results in very different saturation times.

Note the agreement of the theory with other experimental datapoints for shorter timescales, also for those of glucose solution, even though the *c* constant value was determined based only on ribose data after 90 days of incubation.

The other two curves show the prediction of the theory for a glucose concentration of 100 mg dl^−1^ (green), *i.e.* corresponding to the normal glucose concentration in blood which is around 65–110 mg dl^−1^, and for a ribose concentration of 100 mg dl^−1^ (magenta). Even after a long period of 10 years, at a safe concentration of glucose below the IFG of 126 mg dl^−1^ (American Diabetes Association, 2016 ▸), we will have only about 16% of all possible glycations. Conversely, for ribose the situation would be more compromised, with more than 60% of all possible glycations having already occurred.

**Figure 12:**
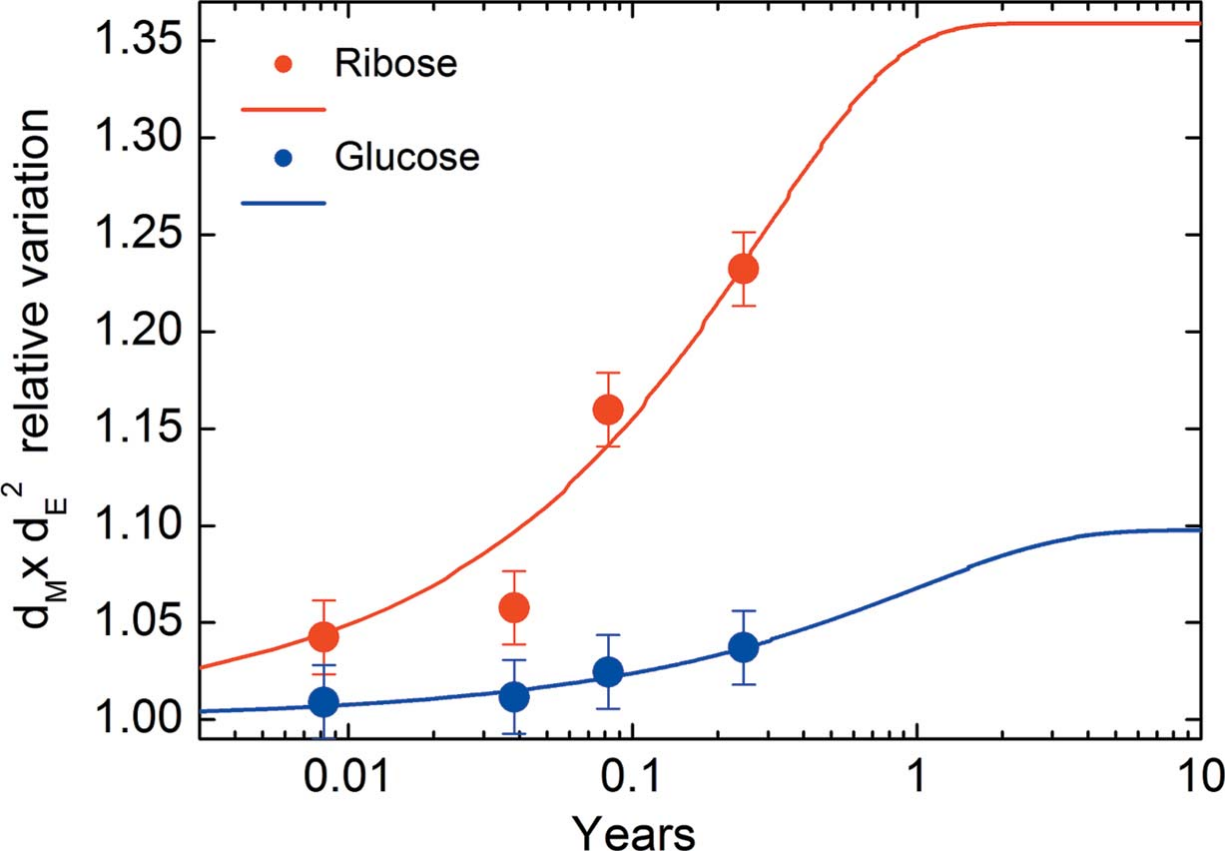
Variation of the volume occupied by glycated collagen with respect to the native molecule for a sugar concentration of 40 mg ml^−1^.

Finally, Fig. 12 ▸ shows the volume occupied by glycated collagen relative to that of the native molecule, in comparison with the experimental values listed in Tables 1 ▸ and 2 ▸.

We found a good agreement between the model and the experimental data and saturation values of 36 and 10% volume increments, for ribose and glucose, respectively. A total of 90% of the maximum relative volume variation (saturation onset) is reached after 0.64 and 2.27 years for the ribose and glucose solutions, respectively. Thus, after 90 days of incubation (*i.e.* after about 0.25 years, at a concentration of 40 mg ml^−1^) we were still far from experimentally observing saturation effects, even for the ribose solution.

## 4. Summary, conclusions and outlook   

Based on the models described in this paper, we determined the parameters characterizing the glycation of collagen upon incubation in glucose and ribose. From the experimental data, we determined the increase of the linear electron density along the collagen fibrils and the volume change due to glycation. The increase in electron density coincides with the positions of the arginine and lysine/hy­droxy­lysine amino acids, which suggests considering these as the primary sites of glycation. Based on the molecular structure of collagen, we estimate the number of sites and the likelihood for glycation. We assume that the topological PSA of the sugar molecules determines the glycation rates. With this input, we model the glycation as a function of time and determine the glycation rate and thus progression of glycation as well as the resulting volume increase.

Using our model based on data after 90 days of incubation to predict the state after a long period of 10 years at a safe concentration of glucose, 100 mg dl^−1^ (*i.e.* below the threshold for IFG of 126 mg dl^−1^), we find that 16% of all possible glycations have taken place. For comparison, in the case of ribose more than 60% of all possible glycations would have taken place after 10 years. These values seem to be consistent with what we found in the literature on comparable, well defined model systems.

The fact that the theoretical descriptions developed in this paper describe the experimental data well is an indication of the underlying assumptions and simplifications being justified and thus recognition of the basic mechanisms. The parameters should be refined in future studies for the case of glucose, as glycation progresses so slowly that the resulting error bars are significantly higher than for ribose.

Our model with refined parameters may be used to estimate the glycation state for realistic progressions of blood sugar concentrations occurring in healthy and diabetic subjects. This is immediately feasible, using realistic daily blood-sugar variations and the parameters and model described in this study as input. This glycation state could be verified against experimental data, taking available information on the vastly varying renewal rates of collagen in its various functions in the body into account. Such a study would be feasible, provided access to tissue from human donors of known diabetic status is available – or from a well characterized and long-lived animal model. For this, the changing degradation and thus renewal rates as a function of the glycation state need to be known. Conversely, such parameters could be verified assuming the correctness of the model and sufficient knowledge of the progression of the blood sugar concentration.

We hope that a better understanding of glycation has been reached and that the model system used for determining the experimental data for this study could potentially be validated as a testbed for glycation and diabetes, without the need for sacrificing animals. Given different sugars, methods complementary to X-ray characterization, such as mass spectrometry, could allow decoupling of the levels of glycation from variations in the structural effects of glycation.

## 5. Experimental   

Sample preparation, X-ray data acquisition and analysis have been described previously (Giannini *et al.*, 2019 ▸, 2021 ▸). Briefly, we analyzed the effect of non-enzymatic glycation on collagen-rich matrices of decellularized bovine pericardium *in vitro*, in order to investigate how the multiscale structure of type I collagen is affected by different monosaccharides, d-glucose, d-galactose and d-ribose, and exposure time. The cattle tissues, kindly provided by Gavazza 1913 Spa (Asti, Italy), were first decellularized by both chemical and enzymatical approaches, and subsequently, for the glycation studies, the matrices were soaked at 37°C in solutions with increasing concentration of d-glucose, d-galactose and d-ribose concentrations [(1) = 0, (2) = 2.5, (3) = 5, (4) = 10, (5) = 20, (6) = 40 mg ml^−1^] and at increasing time [3, 14, 30 and 90 days]. Furthermore, the monosaccharide concentrations in the matrices, for each time point, were evaluated using the proper Assay Kit (Abnova, Germany and Sigma– Aldrich, Italy) for monosaccharides and through fluorescence spectroscopy (Victor X4 Multilabel Plate Reader, Perkin Elmer, Italy) with an excitation wavelength of 540 nm and an emission wavelength of 590 nm. The normalization with weight allowed us to obtain the concentration per milligram of tissue. SAXS and WAXS scanning microscopies were performed on decellularized tissues and data were collected at the cSAXS beamline of the Swiss Light Source in Villigen, Switzerland (Bunk *et al.*, 2009 ▸). The experimental setup described in previous works (Giannini *et al.*, 2019 ▸, 2021 ▸) consists of a liquid N_2_-cooled fixed-exit Si(111) monochromator with a second rotating crystal that can obtain a spot size down to 20 µm FWHM in the horizontal direction, and a mirror to reject higher X-ray energies and for vertical focusing. For SAXS measurements, a 7 m-long evacuated flight and a Pilatus 2M detector were used, while for WAXS data collection the detector was placed close to the sample position. The tissues, previously sterilized in ethanol 70%(*v*/*v*), washed three times in phosphate-buffered saline (PBS) and cut into 1 cm^2^ samples, were placed in Ultralene sachets with a drop of PBS and 0.05% penicillin/streptomycin solution, to avoid deterioration, and then sealed. Samples were placed on the sample holder and moved at a constant speed and step size over the exposure time vertically. 2D SAXS and WAXS data were calibrated with silver behenate and NIST SRM640b, respectively, folded into 1D profiles, and integrated in 16 azimuthal segments. For each sample, 4131 SAXS and 4131 WAXS 2D data frames were recorded.

### 5.1. Fourier difference phasing   

In principle, phasing the square root of the measured SAXS intensities allows us to retrieve the 1D collagen electron density, as a function of the fractional coordinate along the *d*_M_ periodicity. To obtain a solution by any phasing algorithm, suitable boundary conditions or a starting phase, derived by a suitable model, is needed. In turn, this implies a bias of the model on the retrieved solution. In addition, the nature of the SAXS data with small diffraction peaks on a very large 1/*q* ^2^ background adds to the uncertainties of the derived parameters.

The inverse FFT of the full measured SAXS pattern *I*(*q*) produces the Patterson function, related to the auto-correlation function of the unknown electron density. The FFT of *I*(*q*) at zero days of incubation gives the auto-correlation function of the native collagen electron density, namely the auto-correlation of ρ_ini_. In the same way, the FFT of *I*(*q*) after 90 days of incubation of collagen in a sugar solution gives the auto-correlation function of the glycated collagen electron density, namely the auto-correlation of ρ_fin_ = ρ_ini_ + ρ_gl_. The Patterson difference is simply calculated by the two suitably rescaled sets of measured intensities. This readily gives a result related to the autocorrelation of the unknown function ρ_gl_, but with a contribution related also to the convolution of ρ_gl_ with ρ_ini_.

On the contrary, a Fourier difference synthesis provides a direct estimate of ρ_gl_. It can be calculated using the subtracted amplitudes between the SAXS data measured on the glycated samples with respect to native ones, assuming the phases are derived by a suitable model. As phases of the model one could assume those of the native structure (Hadely *et al.*, 2001 ▸). However, this choice is also subjected to a model-biased final result. Indeed, the actual ‘native’ structure (ρ_ini_) is unknown, since we know only the ‘ideal’ type-I collagen structure, because some cross-links due to aging are already present in the native structure analyzed through SAXS characterization.

To avoid these model-dependent limitations, we assumed a step-like function as the starting model, equal to 1 in half the *d*_M_ period and 0 in the complementary region. This is the minimal *a priori* information we can impose on the native structure. This approach reduces the accuracy and the chances of success in retrieving the unknown function ρ_gl_, but the final result is not biased by any assumption about a specific native structure.

As the starting function (step-like function) is very rough, we have realized a phasing approach involving two distinct steps: (i) to retrieve a less rough first estimate of ρ_gl_; (ii) the Fourier difference phasing, to refine this estimate.

#### 5.1.1. First step   

Starting from the phase derived by the step-like function we performed some phasing iterations by imposing, as constraints, the measured moduli in the Fourier space, and the positivity of the electron density in direct space, for both ρ_fin_ and ρ_ini_. We selected only the peaks of the measured SAXS patterns. After each phasing cycle, we averaged the results, obtaining a function ρ_ave_ = 0.5(ρ_fini_ + ρ_ini_) as starting trial for the following iteration. After 90% of the whole iteration cycles, the two trial solutions are not averaged anymore, and the algorithm imposes the two constraints, in the real and Fourier space, independently on the unknown functions ρ_fini_ and ρ_ini_. After 200 iterations this first step of the phasing process allows us to obtain an estimate of ρ_gl_ = ρ_fini_ − ρ_ini_ refined with respect to the input one.

#### 51.2. Second step   

This estimated value for ρ_gl_ was used as input in the Fourier difference phasing, where the constraint in the Fourier space was given by the amplitude being set to the difference of the measured SAXS moduli at 90 and 0 days of incubation of collagen in the sugar solution. The constraint in real space was the positivity of the unknown function to be retrieved. A total of 500 cycles sufficed to reach convergence.

The trial solution obtained by the Fourier difference phasing (second step) can be used as a starting function for a new refinement, by applying again the first step of the phasing algorithm, in turn used as input for a further Fourier difference phasing, and so on, repeating the first and second steps of the phasing algorithm many times. The result obtained after seven cycles of refinement (*i.e.* after seven repetitions of the first and second phasing steps) is shown in Fig. 3 ▸, thus obtained after 7 × (200 + 500) ≃ 5000 total phasing cycles, summing those of the first and second steps.

The modeling and in-depth analysis of the data described in the present article have been implemented in *Mathematica*.

## 6. Related literature   

The following references are cited in the supporting information: Chandross & Bear (1973 ▸); Darros–Barbosa *et al.* (2003 ▸); Wess *et al.* (1998 ▸).

## Supplementary Material

Supporting information. DOI: 10.1107/S2052252521010344/fc5057sup1.pdf

## Figures and Tables

**Table 1:** SAXS/WAXS parameters measured on collagen in a 40 mg ml^−1^ ribose solution

	SAXS	WAXS	Δρ
Incubation time (days)	Meridional period *d*_M_ (nm)	Equatorial period *d*_E_ (nm)	Relative electron density difference between overlap and gap regions
0	65.5 ± 0.1	1.514 ± 0.007	0.055 ± 0.001
3	65.4 ± 0.1	1.547 ± 0.005	0.057 ± 0.001
14	65.0 ± 0.1	1.563 ± 0.005	0.057 ± 0.001
30	64.9 ± 0.1	1.638 ± 0.006	0.060 ± 0.001
90	64.4 ± 0.1	1.695 ± 0.012	0.067 ± 0.001

**Table 2:** SAXS/WAXS parameters measured on collagen in a 40 mg ml^−1^ glucose solution

	SAXS	WAXS	Δρ
Incubation time (days)	Meridional period *d*_M_ (nm)	Equatorial period *d*_E_ (nm)	Relative electron density difference between overlap and gap regions
0	65.5 ± 0.1	1.513 ± 0.006	0.054 ± 0.001
3	65.4 ± 0.1	1.522 ± 0.003	0.052 ± 0.001
14	65.4 ± 0.1	1.524 ± 0.005	0.055 ± 0.001
30	65.5 ± 0.1	1.5325 ± 0.004	0.054 ± 0.001
90	65.4 ± 0.1	1.543 ± 0.006	0.056 ± 0.001

## References

[bb1] Ahmed, N. (2005). *Diabetes Res. Clin. Pract.* **67**, 3–21. 10.1016/j.diabres.2004.09.004 15620429

[bb2] American Diabetes Association (2016). *Diabetes Care*, **39**(Suppl. 1), S13.

[bb3] Bailey, A. J., Sims, T. J., Avery, N. C. & Halligan, E. P. (1995). *Biochem. J.* **305**, 385–390. 10.1042/bj3050385 PMC1136373 7832750

[bb4] Bella, B., Brodsky, B. & Berman, H. M. (1995). *Structure*, **3**, 893–906. 10.1016/S0969-2126(01)00224-6 8535783

[bb5] Bunk, O., Bech, M., Jensen, T. H., Feidenhans’l, R., Binderup, T., Menzel, A. & Pfeiffer, F. (2009). *New J. Phys.* **11**, 123016.

[bb101] Chandross, R. J. & Bear, R. S. (1973). *Biophys. J.* **13**, 1030–1048. 10.1016/S0006-3495(73)86043-6 PMC1484344 4748375

[bb6] Chen, P., Cescon, M. & Bonaldo, P. (2015). *Mol. Neurobiol.* **52**, 216–225. 10.1007/s12035-014-8862-y 25143238

[bb102] Darros–Barbosa, R., Balaban, M. O. & Teixeira, A. A. (2003). *Int. J. Food Prop.* **6**, 195–214.

[bb8] Gautieri, A., Passini, F. S., Silván, U., Guizar-Sicairos, M., Carimati, G., Volpi, P., Moretti, M., Schoenhuber, H., Redaelli, A., Berli, M. & Snedeker, J. G. (2017). *Matrix Biol.* **59**, 95–108. 10.1016/j.matbio.2016.09.001 27616134

[bb9] Gautieri, A., Redaelli, A., Buehler, M. J. & Vesentini, S. (2014). *Matrix Biol.* **34**, 89–95. 10.1016/j.matbio.2013.09.004 24060753

[bb10] Gelse, K., Pöschl, E. & Aigner, T. (2003). *Adv. Drug Deliv. Rev.* **55**, 1531–1546. 10.1016/j.addr.2003.08.002 14623400

[bb11] Giannini, C., De Caro, L., Terzi, A., Fusaro, L., Altamura, D., Diaz, A., Lassandro, R., Boccafoschi, F. & Bunk, O. (2021). *IUCrJ*, **8**, 621–632. 10.1107/S2052252521005054 PMC8256709 34258010

[bb12] Giannini, C., Terzi, A. & Fusaro, L. (2019). *Biophotonics*, **12**, e201900106. 10.1002/jbio.201900106 PMC7065647 31211508

[bb13] Giraud-Guille, M.-M. (1992). *J. Mol. Biol.* **224**, 861–873. 10.1016/0022-2836(92)90567-4 1569562

[bb7] Gkogkolou, P. & Böhm, M. (2012). *Dermato-Endocrinology*, **4**, 259–270. 10.4161/derm.22028 PMC3583887 23467327

[bb16] Hadley, J., Malik, N. & Meek, K. (2001). *Micron*, **32**, 307–315. 10.1016/s0968-4328(00)00032-9 11006510

[bb17] Hadley, J. C., Meek, K. M. & Malik, N. S. (1998). *Glycoconj. J.* **15**, 835–840. 10.1023/a:1006928403140 9870360

[bb19] Hudson, D. M., Archer, M., King, K. B. & Eyre, D. R. (2018). *J. Biol. Chem.* **293**, 15620–15627. 10.1074/jbc.RA118.004829 PMC6177574 30143533

[bb21] León-López, A., Morales-Peñaloza, A., Martínez-Juárez, V. M., Vargas-Torres, A., Zeugolis, D. I. & Aguirre-Álvarez, G. (2019). *Molecules*, **24**, 4031. 10.3390/molecules24224031 PMC6891674 31703345

[bb22] Madhurapantula, E. S. & Orgel, J. P. R. O. (2017). *Acceleration Physics-Radiation Safety and Applications*, edited by I. Ahmad & M. Malek, Chapter 7, *X-ray Diffraction Detects D-Periodic Location of Native Collagen Crosslinks in situ and Those Resulting from Non-Enzymatic Glycation*, https://doi.org/10.5772/intechopen.71022. London: InTechOpen.

[bb23] Orgel, J. P., Wess, T. J. & Miller, A. (2000). *Structure*, **8**, 137–142. 10.1016/s0969-2126(00)00089-7 10673433

[bb24] Orgel, J. P. R. O., Miller, A., Irving, T. C., Fischetti, R. F., Hammersley, A. P. & Wess, T. J. (2001). *Structure*, **9**, 1061–1069. 10.1016/s0969-2126(01)00669-4 11709170

[bb25] Persikov, A. V., Pillitteri, R. J., Amin, P., Schwarze, U., Byers, P. H. & Brodsky, B. (2004). *Hum. Mutat.* **24**, 330–337. 10.1002/humu.20091 15365990

[bb26] Ramachandran, G. N. & Kartha, G. (1955). *Nature*, **176**, 593–595. 10.1038/176593a0 13265783

[bb27] Sell, D. R., Biemel, K. M., Reihl, O., Lederer, M. O., Strauch, C. M. & Monnier, V. M. (2005). *J. Biol. Chem.* **280**, 12310–12315. 10.1074/jbc.M500733200 15677467

[bb103] Wess, T. J., Hammersley, A. P., Wess, L. & Miller, A. (1998). *J. Mol. Biol.* **275**, 255–267. 10.1006/jmbi.1997.1449 9466908

