# Supplementary material for: Time scale of glycation in collagen of bovine pericardium-derived bio-tissues
Source: IUCrJ. 2021 Oct 28;8(Pt 6):1024–34. doi: 10.1107/S2052252521010344 (PMC8562672; doi:10.1107/S2052252521010344)
Supplement: Supplementary file 1 [file m-08-01024-sup1.pdf]

IUCrJ

Volume 9 (2022)

Supporting information for article:

Time scale of glycation in collagen in bovine pericardium derived bio-tissues

Liberato De Caro, Alberta Terzi, Luca Fusaro, Davide Altamura, Francesca Boccafoschi, Oliver Bunk and Cinzia Giannini

Time scale of glycation in collagen

Liberato De Caro[a,*], Alberta Terzi[a1], Luca Fusaro[bc1], Davide Altamura[a], Ana Diaz[d], Francesca Boccafoschi[ab], Oliver Bunk[d*] and Cinzia Giannini[a,*]

[a]Institute of Crystallography, National Research Council, via Amendola 122/O, Bari, 70126, Italy

[b]Department of Health Sciences, University of Piemonte Orientale, Novara, Italy

[c]Tissuegraft srl., Novara, Italy

[d]Paul Scherrer Institut, 5232, Villigen PSI, 5232, Switzerland

Correspondence email: liberato.decaro@ic.cnr.it, oliver.bunk@psi.ch, cinzia.giannini@ic.cnr.it

[1]These authors contributed equally to this work.

Funding information Ministero Università Ricerca (grant No. 2017CBHCWF).

Number of sugar molecules involved in the glycation

To calculate more quantitatively the number of sugar molecules involved in the glycation, per staggered repetition unit, we computed the relative electron density values by means of the following relation (1):

$$\Delta\rho = \frac{1}{5} \times \frac{\rho_c - \rho_s}{\rho_c} \qquad \text{(S1)}$$

Here, the absolute electron density values of the collagen ρ_c and the solution ρ_s are expressed in terms of # el./ Å^3. The factor 5 is because the gap region, $(1-\sigma)\times d_M$, contains one gap and four' overlapping sections; this region is alternated to the denser zones, $\sigma\times d_M$, made of five overlapping sections, without any gap, as schematically shown in Figure 1.

To determine the absolute electron density values for the case under study, we have reported all the relevant physical properties, both for collagen and for the ribose and glucose in Table S1. Using theoretical interpolation formulae, the volume of the solution of 40 g of sugar and 1 L of water could be approximated with 1.014±0.001 L for all sugars used here (2). Measures of volume's variations of 1 mL of water, with added 39.9±0.1 mg of ribose and 40.4±0.1 mg of glucose have

given, respectively, 1018±1 μL and 1010±1 μL. Thus, the volumes of the solutions of 1 L of water and 40 g either of ribose or glucose, are 1.018±0.001 L, 1.010±0.001 L, respectively.

Table S1. Physical properties of ribose and collagen needed to calculate the electron density seen by X-rays. N_A is the Avogadro Number. In particular, the absolute electron density values of the last column are obtained from those reported in the second-last, multiplying them by the Avogadro Number N_A and converting the volume in Å^3. In turn, the second-last column's values are determined by the number of moles/volume (sixth column) times Ne (the number of electrons/molecules in the fourth column).

	**Mass Density (gr/cm^3)**	**Mole Mass (gr)**	**N_e=# el. / molecule**	**Volume of 1 mole (Å^3)**	**Mole/L**	**# el./L**	**Electron Density # el./ Å^3**
**Ribose**	0.80	150.13	80	1.88×10^{26}	5.33	426.4×$\mathbf{N_A}$	0.256
**Glucose**	1.54	180.156	96	1.17×10^{26}	8.55	820.8×$\mathbf{N_A}$	0.494
**Water**	1.00	18.016	10	1.8×10^{25}	55.51	555.1×$\mathbf{N_A}$	0.334
**Collagen**	1.45	300000 (3,4)	160000	2.07×10^{29}	0.00483	772.8×$\mathbf{N_A}$	0.465 (ρ_c)
**Solution of Ribose (*)**	1.022	/	/	/	54.53×H_2O +0.262×Rib	566.3×$\mathbf{N_A}$	0.341 (ρ_s)
**Solution of Glucose (*)**	1.030	/	/	/	54.96×H_2O +0.220×Glu	570.7×$\mathbf{N_A}$	0.344 (ρ_s)

(*) 40 mg/mL in 1 L of H_2O.

For ribose, we obtain:

$$\Delta\rho_i = \frac{1}{5}\times\frac{\rho_c-\rho_s}{\rho_c} = \frac{1}{5}\times\frac{0.465-0.341}{0.465} = 0.053\pm0.001 \qquad \text{(S2)}$$

For glucose: $\Delta\rho_i = 0.052\pm0.001$. These relative electron density values agree with the initial relative electron density values, derived from the SAXS experimental data: 0.055 ± 0.001 for the ribose solution and 0.054 ± 0.001 for the glucose solution.

To estimate how many sugar molecules are involved in the glycation process, we need to evaluate the variation of the volumes V_G and V_O of the gap and overlap zones in the collagen structure, respectively. This variation can be estimated by approximating this volume with a cylinder of height $5\times d_M$ and diameter d_E, as schematically shown in Figure 1, and by imposing that the relative electron density's changes must be equal to the values previously determined:

$$(V_G)_{i/f} = \pi\times(1-\sigma_{i/f})\times d_{Mi/f}\times\left(\frac{d_{Ei/f}}{2}\right)^2 , \quad (S3)$$

$$(V_O)_{i/f} \equiv \pi\times\sigma_{i/f}\times d_{Mi/f}\times\left(\frac{d_{Ei/f}}{2}\right)^2 , \quad (S4)$$

$$\frac{\rho_c\times(V_G)_i+\Delta e}{(V_G)_f}\times(1-5\times\Delta\rho_f) - \frac{\rho_s\times(V_O)_i}{(V_O)_f} = 0 . \quad (S5)$$

Here the subscripts "i" and "f" denote initial (day=0) and final (days=90) values. Δe is the number of electrons needed to explain the changes in the relative electron density due to sugar molecules involved in the glycation. Eq. (S4) can be readily derived by Eq. (S2) and it is, in fact, a generalization for $\Delta\rho_f$., obtained after the incubation of collagen in the sugar solution.

We have determined the difference of the electron density in the overlapping region, averaged on the five nanofibrils, with respect to the average value in the gap region, averaged on four nanofibrils only. Therefore, denoted with N the average number of sugar molecules involved in the glycation process in the whole collagen staggered repetition unit, we can put in Eq. (S5) $\Delta e \cong N\times N_e$, obtaining:

$$N \cong \frac{1}{N_e}\left[\frac{1}{(1-5\times\Delta\rho_f)}\times\frac{(V_G)_f}{(V_O)_f}\times(V_O)_i\times\rho_s - (V_G)_i\times\rho_c\right] . \quad (S6)$$

From

$$\frac{(V_G)_f}{(V_O)_f} = \frac{1-\sigma_f}{\sigma_f} , \quad (S7)$$

we finally have

$$N \cong \frac{\pi\times d_{Mi}}{N_e}\times\left(\frac{d_{Ei}}{2}\right)^2\times\left[\frac{1}{(1-5\times\Delta\rho_f)}\times\frac{1-\sigma_f}{\sigma_f}\times\sigma_i\times\rho_s - (1-\sigma_i)\times\rho_c\right] . \quad (S8)$$

For $\sigma_f = \sigma_i = \sigma$, Eq. (S8) becomes:

$$N \cong \frac{\pi \times d_{Mi}}{N_e} \times \left(\frac{d_{Ei}}{2}\right)^2 \times (1-\sigma) \times \left[\frac{\rho_s}{(1-5\times\Delta\rho_f)} - \rho_c\right], \qquad \text{(S9)}$$

plotted in Figure 8.

In-plane packing areal density of glycated collagen molecules

Given the sugar concentration m/V, we want to evaluate how many sugar molecules are contained in a cylindrical volume of solution comparable to the volume of a collagen staggered repetition unit, i.e., of diameter d_E=1.5 nm and length d_M=5×65.5 nm:

$$N_{sugar} = \frac{m}{M} \times \frac{N_A}{V} \times 5\pi \left(\frac{d_E}{2}\right)^2 \times d_M \qquad \text{(S10)}$$

Here, V is the volume of the solution, m the mass of the solute, M its mole mass, N_A the Avogadro number. This number of molecules is constant during the incubation time, because the sugar solution has been weekly refilled. After 90 days of incubation for the ribose solution we found d_E=1.695 nm and d_M=64.4 nm (see Table 1), to which corresponds, based on Eq. (S10), $N_{rib}(90\ \text{days})$=114.5±1.5 ribose molecules. The same calculation for glucose gives, after 90 days: $N_{glu}(90\ \text{days}) =$ 80.7±1.5. At 0 days we have $N_{rib}(0\ \text{days})$ =91.2±1.5 and $N_{glu}(0\ \text{days})$ =76.6±1.5. The increment of about 3 sugar units for glucose and 22 units for ribose, given by Eq. (S10), are related to the increment of the volume in the 90 days of incubation of collagen in the solution.

The topological polar surface area (PSA) are PSA_{rib}=90.2 Å^2, for ribose, and PSA_{glu}=110 Å^2, for glucose. Assuming a circular surface, this implies a topological polar mean size of about 0.54 nm, for ribose, and about 0.59 nm for glucose. Therefore, the lateral empty space between collagen molecules could be enough to accommodate more easily the smaller ribose molecule with respect to the glucose ones, explaining difference of glycation rates experimentally measured on different sugars in solution.

To quantify this aspect, Figure S1 shows a schematic drawing of a standard in-plane packing, with the collagen molecules aligned along the three principal planes of molecular packing (5) but allowing enough space to accommodate ribose molecules (green circles). The circles have been drawn with a size proportional to the actual ratio of molecules. In other words, the ratio of the red and green circles is equal to 1.51/0.54=0.28. When we put in contact these circles, we impose a planar geometrical constraint. If we put in contact red circles (collagen molecules) and green circles (ribose molecules) we found that the rectangular blue box of Figure 9, which contains five aligned

collagen molecules, has a size which is 5.5 that of the red circle, 10% larger than the size of 5 collagen in-contact molecules. This simple geometrical model, of circles in contact, leads to a 10% larger space between collagen molecules (red circles), to allow the presence of green circles (ribose molecules). This lateral spacing variation is comparable to the increase in the measured lateral distance between nanofibrils, experimentally found from WAXS (Table 1): 100×(1.695-1.514)/1.514=12%. Therefore, the geometrical constraint of circles in contact would allow a first rough estimate of in-plane packing areal density of the structure. Indeed, the area of 5 red circles (5 collagen molecules) divided the area of 1 unit cell is equal to 0.68, would give the in-plane packing areal density of collagen molecules, in a ribose solution of 40 mg/mL, after 90 incubation days.

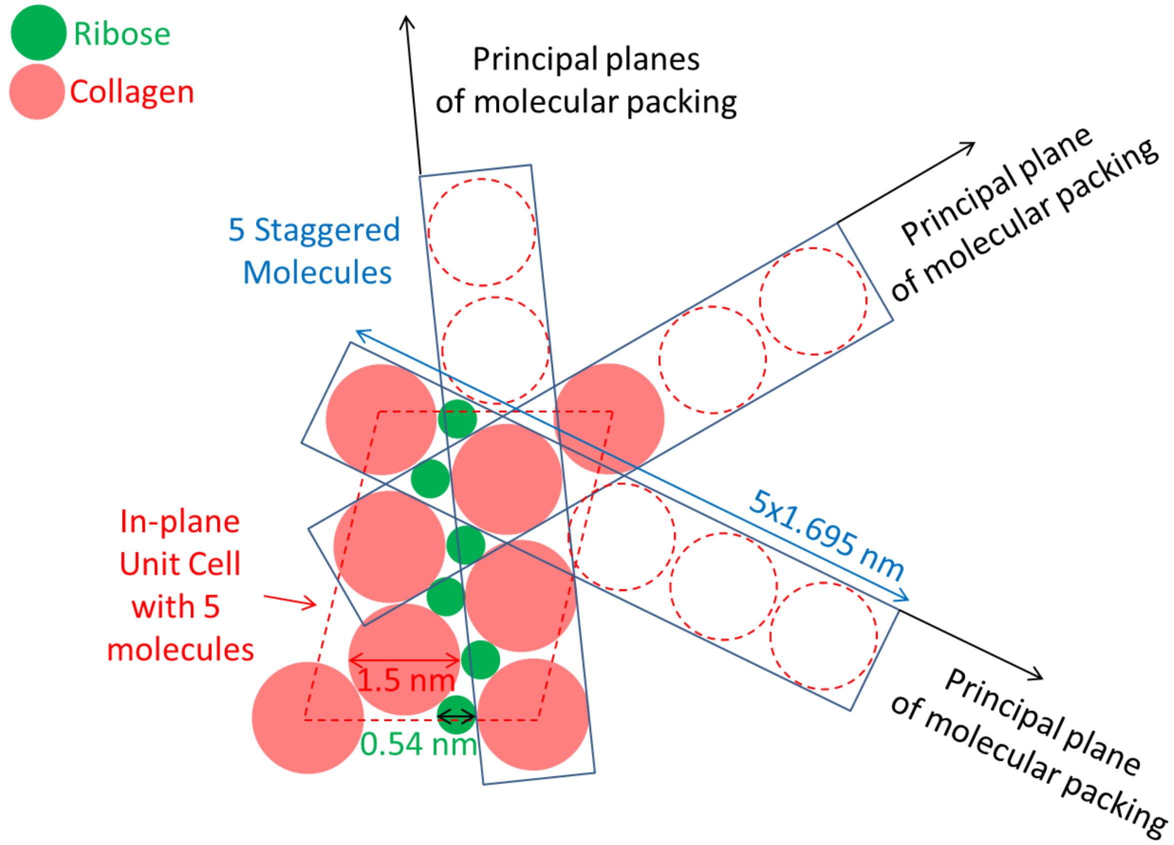

Figure S1. Schematic draw of a standard in-plane packing, with the collagen molecules aligned along the three principal planes of molecular packing, showing some ribose molecules (green molecules) between collagen molecules (red circles). The scale for this drawing is given by the diameter of the red circles (collagen) being 1.5 nm.

To be more quantitative, for an arbitrary lateral packing of cylinders, we will denote with *w* the fraction of space between cylinders, complementary to the in-plane packing areal density. After 90

days we have measured that 36.8±5.7 ribose molecule per staggered repetition unit. Thus, we can estimate *w* by the following relation:

$$w_{rib}(90) = \frac{36.8\pm5.7}{114.5\pm1.5}=0.32\pm0.05 \qquad \text{(S11)}$$

in agreement with the previous estimate, based on the geometrical model sketched in Figure 9, since 1-0.68=0.32.

The maximum (2D hexagonal lattice) density packing of cylinders, in contact with each other, would fill about 91% of the available space. It is known that collagen molecules in plane are not characterized by an optimal spacing in a quasi-hexagonal array (5), thus showing smaller packing areal density than the maximum possible value. We found 68% after ribose-glycation. However, the squared relative variation of d_E equatorial spacing varies linearly as a function of the incubation time (see Figure 9). Given the linear relation deduced by Figure 9, the fraction of space between collagen molecules after 90 days of incubation in a ribose solution of 40 mg/mL, is about 26% larger than the initial value at *t*=0 days which, consequently, is $w_{nat} = w(0)$=0.255±0.04, leading to a native value of the lateral areal packing of collagen molecules – without glycation – of about 1-w_{native} =0.745±0.04. This value implies a 2D native density packing of about 75%, confirming the not-optimal in-plane spacing of collagen molecules (5).

The fraction of space between cylinders after 90 days, in presence of glucose, for the geometrical model shown in Figure S1, should be only 4% larger than the initial value, at 0 days, giving $w_{glu}(90) = 0.265\pm0.05$. Indeed, the time scale for the d_E variation in presence of a solution of 40 mg/mL of glucose is 1/Sqrt[*f*]=1/Sqrt(38)≅0.16 of that deduced for a 40 mg/mL ribose solution, as shown in Figure 9.

Therefore, from the linear fit of Figure 9, we can deduce:

$$w_{rib}(t)\cong\left(1+\sqrt{\Delta d_E\times t}\right)^2\times w_{nat} \qquad \text{(S12)}$$

$$w_{glu}(t)\cong\left(1+\sqrt{\Delta d_E\times t/f}\right)^2\times w_{nat}. \qquad \text{(S13)}$$

Another fraction of the cylindrical volume, that can be associated to a collagen staggered repetition unit, available for sugar molecules, is the gap region. It is equal to about 1/10≅(1-σ)/5. Thus, the total effective volume available for sugar molecules will be:

$$w_{rib}(t)\cong\frac{1-\sigma}{5}+\left(1+\sqrt{\Delta d_E\times t}\right)^2\times w_{nat} \qquad \text{(S14)}$$

$$w_{glu}(t)\cong\frac{1-\sigma}{5}+\left(1+\sqrt{\Delta d_E\times t/f}\right)^2\times w_{nat}. \qquad \text{(S15)}$$

Density variations along the axis of collagen molecules

Also the squared relative variation of d_M meridional period varies linearly as a function of the incubation time. The fitting line of the ribose data, shown in Figure 10, has the following slope: $\Delta d_M = -3.1\times10^{-6} \pm 0.15\times10^{-6}$ days^{-1}. Thus Δd_M is two order of magnitude smaller than Δd_E. Consequently, even at a concentration of 40 mg/mL, any variation on the glucose data is not appreciable after 90 days of incubation, if we assume a factor f also for the time scale of the d_M variation in presence of a solution of 40 mg/mL of glucose, with respect to the 40 mg/mL ribose solution's case. Indeed, it is interesting to note that also the squared relative variation of d_M meridional period varies linearly as a function of the incubation time. This finding allows to write the following relation:

$$d_{M,rib}(t) \cong \left(1 - \sqrt{\Delta d_M \times t}\right) \times d_{M,rib}(0). \quad \text{(S16)}$$

If we assume for glucose

$$d_{M,glu}(t) \cong \left(1 - \sqrt{\Delta d_M \times t/f}\right) \times d_{M,glu}(0), \quad \text{(S17)}$$

after 90 days of incubation, from Eq. (S17) it follows that we have a variation of the $d_{M,glu}(t)$ period of only 0.3%, i.e., of about 0.18 nm, too close to the experimental error of ±0.1 nm to be well evidenced by experiments, as shown in Figure 10.

Also the variation of the meridional period contributes to the total variation of the effective fraction of volume available for sugar molecules, between collagen molecules. Thus, taking into account also the density variations along the collagen molecule axis, Eqs. (S14) and (S15) can be generalized as follows:

$$w_{rib}(t) \cong \frac{1-\sigma}{5} + \left(1 + \sqrt{\Delta d_E \times t}\right)^2 \times \left(1 - \sqrt{\Delta d_M \times t}\right) \times w_{nat} \quad \text{(S18)}$$

$$w_{glu}(t) \cong \frac{1-\sigma}{5} + \left(1 + \sqrt{\Delta d_E \times t/f}\right)^2 \times \left(1 - \sqrt{\Delta d_M \times t/f}\right) \times w_{nat}. \quad \text{(S19)}$$

denoted in the main text as $w_{sugar}(t)$, meaning Eq. (S18) for ribose and (S19) for glucose.

References

1. R. J. Chandross and R. S. Bear, Improved profiles of electron density distribution along collagen fibrils, Biophysical J. 13, 1973, 1030-1048.

2. Roger Darros Barbosa, Murat O. Balaban & Arthur A. Teixeira, Temperature and Concentration Dependence of Density of Model Liquid Foods, International Journal of Food Properties 6 (2003), 195-214, DOI: 10.1081/JFP-120017815.

3. Gelse K., Pöschl E., Aigner T. Collagens—structure, function, and biosynthesis. Adv. Drug Deliv. Rev. 2003;55:1531–1546. doi: 10.1016/j.addr.2003.08.002.

4. León-López A., Morales-Peñaroza A., Martínez-Juárez V.M., Vargas-Torres A., Zeugolis D.I., Aguirre-Alvarez G. Hydrolized collagen—Sources and Applications. Molecules. 2019;24:4031. doi: 10.3390/molecules24224031.

5. T. J. Wess1, A. P. Hammersley, L. Wess and A. Miller. Molecular Packing of Type I Collagen in Tendon, J. Mol. Biol. (1998) 275, 255-267.
